# A qualitative study of the drivers of socioeconomic inequalities in men’s eating behaviours

**DOI:** 10.1186/s12889-018-6162-6

**Published:** 2018-11-14

**Authors:** Lena D. Stephens, David Crawford, Lukar Thornton, Dana Lee Olstad, Philip J. Morgan, Frank J. van Lenthe, Kylie Ball

**Affiliations:** 1 Institute for Physical Activity and Nutrition (IPAN), School of Exercise and Nutrition Sciences, Deakin University, Geelong, Australia; 2 Department of Community Health Sciences, Cumming School of Medicine, University of Calgary, 3280 Hospital Drive NW, Calgary, AB Canada; 3 Priority Research Centre in Physical Activity and Nutrition, School of Education, University of Newcastle, Newcastle, New South Wales Australia; 4 Department of Public Health, Erasmus MC, Erasmus University Medical Center, Rotterdam, The Netherlands

**Keywords:** Socioeconomic inequalities, Men, Eating behaviours, Nutrition

## Abstract

**Background:**

Men of low socioeconomic position (SEP) are less likely than those of higher SEP to consume fruits and vegetables, and more likely to eat processed discretionary foods. Education level is a widely used marker of SEP. Few studies have explored determinants of socioeconomic inequalities in men’s eating behaviours. The present study aimed to explore intrapersonal, social and environmental factors potentially contributing to educational inequalities in men’s eating behaviour.

**Methods:**

Thirty Australian men aged 18–60 years (15 each with tertiary or non-tertiary education) from two large metropolitan sites (Melbourne, Victoria; and Newcastle, New South Wales) participated in qualitative, semi-structured, one-on-one telephone interviews about their perceptions of influences on their and other men’s eating behaviours. The social ecological model informed interview question development, and data were examined using abductive thematic analysis.

**Results:**

Themes equally salient across tertiary and non-tertiary educated groups included attitudes about masculinity; nutrition knowledge and awareness; ‘moralising’ consumption of certain foods; the influence of children on eating; availability of healthy foods; convenience; and the interplay between cost, convenience, taste and healthfulness when choosing foods. More prominent influences among tertiary educated men included using advanced cooking skills but having relatively infrequent involvement in other food-related tasks; the influence of partner/spouse support on eating; access to healthy food; and cost. More predominant influences among non-tertiary educated men included having fewer cooking skills but frequent involvement in food-related tasks; identifying that ‘no-one’ influenced their diet; having mobile worksites; and adhering to food budgets.

**Conclusions:**

This study identified key similarities and differences in perceived influences on eating behaviours among men with lower and higher education levels. Further research is needed to determine the extent to which such influences explain socioeconomic variations in men’s dietary intakes, and to identify feasible strategies that might support healthy eating among men in different socioeconomic groups.

**Electronic supplementary material:**

The online version of this article (10.1186/s12889-018-6162-6) contains supplementary material, which is available to authorized users.

## Background

Men tend to eat less healthfully than women, eating fewer fruits and vegetables [1–3], more red and processed meat [2, 4], and greater amounts of processed discretionary foods [3–5]. These differences contribute to gender inequalities across a range of adverse health outcomes including obesity [6], diabetes mellitus [7] and coronary heart disease [8].

Socioeconomic inequalities in diet are well established [9–11]. Men and women experiencing socioeconomic disadvantage (e.g. those with low education, low income, or residing in deprived areas) tend to have eating behaviours not conducive to good health [9–11]. Compared with more advantaged adults, those who are disadvantaged tend to eat fewer fruit and vegetables [9, 12], and less fibre [9]. Disadvantaged adults also consume more fat, skip breakfast [9] and eat fast food more frequently [13].

Education was selected as an indicator of socioeconomic position because it is a strong determinant of future occupation and income, reflects knowledge-related assets and other intellectual resources, and has been strongly associated with dietary intake in previous studies [14].

The social ecological model, which recognises that individuals are embedded within larger social systems, provides a useful framework for investigating determinants of behaviour. According to the model, behaviours are determined by the interactions of individuals and their social and physical environments [15]. While correlates of women’s eating behaviours are well characterized [16–20], influences on men’s eating behaviours are less well understood, and are likely to differ from those that influence women [21, 22]. While some sex difference in intakes may be attributable to biological factors, it is likely that a range of other factors at the individual, social and environmental levels are also implicated. For instance, social norms related to masculinity may lead men to perceive that consumption of certain healthy foods, and activities such as meal planning and cooking are feminine [23], and hence ‘unmasculine’ [24].

Several potential drivers of socioeconomic inequalities in men’s eating behaviours have been identified in studies focussed on singular domains of the social ecological model. Intrapersonal factors including nutrition-related knowledge [25–27], self-confidence, problem-solving skills and the ability to process information are important for helping individuals overcome obstacles to adopting more favourable eating behaviours [25]. Socioeconomically disadvantaged men may also be less likely to use nutrition information and may also lack the skills or confidence to prepare healthy meals [27, 28].

Social norms, particularly those related to masculinity, may also contribute to socioeconomic differences in eating behaviours. Men who endorse dominant norms of masculinity were shown to adopt less optimal eating behaviours than their peers who endorse less traditional norms [23]. Young blue-collar male workers tended to show little consideration for being health-conscious, resulting in consumption of diets high in saturated fats and sugars [29]. Those men’s food practices reflected gender identity, with food preparation commonly viewed as “women’s work”. Blue-collar workers’ food choices were also influenced by poor dietary role models, including peers, co-workers, and supervisors [29].

Environmental factors may also explain socioeconomic differences in men’s eating behaviours, such as differential access to stores selling both healthy and less healthy foods [30]. Disadvantaged men may be less likely to make optimal food choices due to limited access to affordable nutritious foods within the local environments where they work and live. Danish men with low education believed their weight gain was partly attributable to the types of foods available in their work environment [31]. In New Zealand, the least deprived areas had 76% fewer fast food outlets than the most deprived areas, and fast food outlet exposure was negatively associated with individual-level SEP indicators (highest educational attainment and relative income) [30].

## Methods

To our knowledge, potential explanations of socioeconomic differences in men’s eating behaviours across intrapersonal, social and environmental domains have not previously been investigated simultaneously. Further, as these influences remain unexplored across multiple domains together, such an approach may have yielded a better understanding of the interaction between factors from different domains, as well as potentially identifying factors that may have been overlooked when domains were previously investigated in isolation. How these factors may influence socioeconomic inequalities in eating behaviours among men remains unclear. The present investigation aimed to qualitatively explore potential explanations for socioeconomic differences in men’s eating behaviours among men with tertiary and non-tertiary education.

This study is reported according to the consolidated criteria for reporting qualitative research guidelines [32], and was conducted in conjunction with an independent social and market research agency, Market Solutions P/L (http://www.marketsolutions.com.au/). The agency was selected to assist with the study given their strong track record in conducting social science research [33, 34], and their familiarity with qualitative methodology and research, particularly amongst socioeconomically disadvantaged groups. The agency is accredited to the international ISO standard for market, social and opinion research (AS ISO 20252) and is a member of the Association of Market Research Organisations (AMSRO). Market solutions P/L was responsible for recruitment, conducting interviews, recording and transcribing data, and transmitting de-identified data to the study investigators. The study investigators were responsible for all other aspects of the study.

The study was approved by the Deakin University Faculty of Health Human Ethics Advisory Group (HEAG-H; approval HEAG-H 95_2015). All men provided informed, verbal consent to participate. This was recorded by interviewers at the point of first contact with the men in a password protected project database stored on a secure server.

### Participants

The sample comprised 30 men of working age (18–60 years), 15 with a non-tertiary level education, i.e. completed Year 9 or less, Year 10, Year 11, Year 12 (final year of high school in Australia), or Certificate/Diploma/Advanced Diploma; and 15 with a tertiary education level (completed tertiary education, i.e. a Bachelor degree or higher) from Melbourne, Victoria, and Newcastle, New South Wales (large metropolitan regions in two Australian states). To reflect SEP in nutrition research, education is often stratified as described above (high SEP is indicated by having achieved tertiary level qualifications, while low SEP is reflected by achieving non-tertiary level qualifications) [35–37]. The current qualitative data can be used to generate hypotheses that could be followed up in future research [38]. Education was employed as the measure of SEP in this study as it is a relatively stable indicator of SEP [14, 39]. Seven or eight men each with tertiary or non-tertiary education participated from each site. Men of working age were the focus of the present investigation as different factors influencing eating behaviours might be reflected among older men (e.g. those who are retired), given substantial lifestyle changes that come with older age (e.g. income, available time, household structure, health issues [40]).

### Recruitment procedure

Market Solutions P/L accessed telephone directories of community members in both target catchment areas, including mobile and landline numbers and randomly selected men’s numbers to be called by one of three male interviewers (agency employees trained in qualitative methodology). Male interviewers were chosen to maximise the potential to build reciprocity between the interviewer and participant which may yield richer data than may have been gathered by female interviewers [41]. Men were invited to complete a telephone-based interview either immediately or at a more convenient time. Purposive sampling [42] based on educational attainment and city of residence was used to recruit a total of 30 men (15 from each target catchment, and 15 each with tertiary and non-tertiary education).

Interested participants received study information via telephone and were assessed for eligibility (i.e. 18–60 years of age, were tertiary or non-tertiary educated as defined above, and could communicate clearly in English). Men were offered an AUS$20 voucher to a leading retailer as compensation for their time (mailed post-interview).

### Semi-structured interview schedule and procedure

Development of questions for the semi-structured interviews was informed by the social ecological model [15], and previous research examining determinants of men’s eating behaviours [21, 23, 24, 26, 28, 29, 31, 43–46].

Questions were primarily open-ended and aimed at assessing participants’ usual eating behaviours and perceived influences on these (Additional file 1: Table S1). Men were prompted to discuss food task responsibilities; influences on eating behaviours and eating choices (including an exploration of trade-offs between health, convenience, peer modelling, price, accessibility, and taste); body weight; masculinity; social influences; perceptions of other men’s eating behaviours (social norms); and neighbourhood availability of healthy foods.

### Interview procedure

Interviews were conducted by telephone in 2015. A one-on-one telephone interview was chosen as men resided across a wide geographical area making face-to-face interviews less feasible. The interview schedule was pilot tested and refined with the first two men (one with tertiary education from Melbourne, one with non-tertiary education from Newcastle). Piloting showed no major issues with timing or questions, with only minor changes made for clarification. Pilot data were not included in further analyses.

Before commencing, interviewers asked for permission to digitally record the interview, and participants answered sociodemographic questions. Interviews lasted between 25 and 35 min, and once complete, were transcribed verbatim from the recordings.

### Sociodemographic characteristics

Men provided their age (five response categories ranging from 18-24y to 55-60y) and highest attained level of education (six categories ranging from Year 9 or less to Bachelor degree or higher). Employment status (working full-time/part-time, studying, unemployed, retired, home duties, or other), annual household income (eight response categories ranging from <AUS$20,000 to ≥AUS$150,000, and including don’t know, and refused), household structure (couple with children, couple without children, single parent, single person, or flatmates) and occupation (comprising professional, technician/trades worker, community and personal services worker, manager, clerical and administrative worker, machinery operator/driver, sales worker, labourer, and other) were also established.

### Data analysis

Qualitative description was used to build a comprehensive understanding of socioeconomic differences in influences on men’s eating behaviours. Qualitative description aims to maximise descriptive and interpretive validity by providing an account of events (including meanings participants attribute to those events) that both participants and researchers would agree is accurate [47, 48]. This methodology is more appropriate than those requiring a greater degree of researcher interpretation given the goal of the present investigation to discern potential influences on socioeconomic differences between men’s eating behaviours [48].

Data were analysed by the lead author (LS) using thematic analysis, which comprised four key steps [49]: immersion in the data, line-by-line coding, creating categories, and generation of themes. LS read and re-read transcribed interviews to build familiarity with the data (data immersion), and then performed abductive thematic analysis [50] to code data using descriptive labels. Categories were formed by linking coded data together that related to similar concepts, while keeping categories for tertiary and non-tertiary educated men separate [49]. Based on these categories, LS identified key emerging themes that were salient for men within each education level group. Individual influences were each classified into a separate theme (e.g. ‘cost’, ‘convenience’, etc.). Findings were generated via an iterative, abductive cycle, moving back and forth between inductive and deductive reasoning. Where relationships between themes and/or sub-themes were identified, such interactions were classified under the predominant theme that united those factors (e.g. interplay between cost, convenience, taste, and healthfulness of food was described within the ‘cost’ theme. Of these factors, cost was determined to be most predominant as participants typically described cost before discussing consideration of the other factors).

Rigour was maintained via researcher reflexivity (i.e. ensuring one’s own perspectives are left out of the coding process as much as possible), development of an audit trail by recording steps taken in the development and reporting of findings, linking interpretations with the raw data by presenting participant quotes, and peer debriefing with the study’s co-authors throughout the analytical process. An independent researcher (non-author) double-coded a subsample of interviews (20%; *n* = 6, three from each education group). Each coder independently and systematically employed the iterative, abductive cycle described above to create categories from the data. The purpose of double coding was to explore potential alternative interpretations of the data, as the iterative process of cross-checking coding strategies and data interpretation by the researchers enables potential alternative interpretations to be identified and discussed, serving to create a more thorough examination of the data [51]. Data analysis was conducted using raw transcripts entered into NVivo software (version 10, QSR International, Melbourne, Australia).

## Results

Sociodemographic characteristics of the sample are shown in Table 1. A range of age groups were represented, with the majority aged 45–54 years, and employed in full- or part-time work (80% of non-tertiary educated men, 87% of tertiary educated men). Very few men were studying (*n* = 2), unemployed (*n* = 1), or retired (*n* = 1); and none were engaged in home duties or other forms of employment (data not shown). Most tertiary educated men worked as professionals (77%). Among non-tertiary educated men, 50% worked as technicians and trades workers, 17% worked as managers, and 17% in clerical and administrative roles. Only one man was employed as a machinery operator/driver, and none were employed as sales workers, labourers, or in other roles (data not shown).

**Table 1:** Sociodemographic characteristics of participating men

	Socioeconomic position^a^		
	All, n	High SEP, n	Low SEP, n
N	30	15	15
Age (y)			
18–24	1	0	1
25–34	3	2	1
35–44	5	3	2
45–54	17	7	10
55–60	4	3	1
Location			
Melbourne	15	7	8
Newcastle	15	8	7
Employment status			
Work full-time/ part-time	25	13	12
Student	2	1	1
Unemployed	2	1	1
Retired	1	0	1
Occupation^b^			
Professional	10	10	6
Technician/trades worker	7	1	1
Community and personal services worker	3	2	2
Clerical and administrative worker	2	0	2
Manager	2	0	1
Machinery operator/driver	1	0	0
Household income, per annum			
Low (< AUS$60,000)	6	0	6
Mid (AUS$60,001-AUS$100,000)	12	7	5
High (> AUS$100,000)	10	7	3
Don’t know/refused	2	1	1
Household structure			
Couple with children	18	9	9
Couple without children	4	3	1
Single parent	1	0	1
Single person	3	1	2
Flatmates	4	2	2

^a^Highest attained level of education was the main determinant of SEP

^b^Missing for *n* = 5 (*n* = 2 studying, *n* = 2 unemployed, *n* = 1 retired)

Major emerging themes and exemplary quotes are presented below, with results presented stratified by education level. Themes found to be equally prominent across both groups of men included the intrapersonal-level influences of attitudes relating to masculinity, nutrition knowledge and awareness, and ‘moralising’ consumption of certain foods; and social influences of children. Environmental themes discussed by both groups included availability of and access to healthy and unhealthy foods; convenience; and the interplay between cost, convenience, taste and healthfulness when choosing foods (discussed within the cost theme).

Intrapersonal influences more frequently discussed by tertiary educated men within themes identified included having greater food-related skills (e.g. cooking involving multiple, complex steps), but less involvement in food-related tasks (e.g. menu-planning, purchasing) because of time constraints. Almost all tertiary educated men with partners identified their partners as a positive influence on eating behaviours. Environmental influences more dominant among tertiary educated men included accessibility of healthy foods; and perceiving healthy foods as expensive and unhealthy foods as inexpensive.

A number of influences within themes were more frequently discussed by non-tertiary educated men, including having less developed cooking skills but regular involvement in food-related tasks such as shopping, preparing, and cooking meals when compared to discussion by tertiary educated men. While men from both groups recognised nutrition knowledge as an influence on their eating behaviours, non-tertiary educated men reported lower perceived levels of nutrition knowledge, and sometimes described misperceptions related to nutrition and body weight. A theme identified only among non-tertiary educated men was the perception that no-one influenced their eating behaviours. Non-tertiary educated men also identified mobile worksites (i.e. moving from one work location to another during the day/week as necessitated by their job, common among those working as tradesmen) as an unhealthy influence on eating, and discussed the need to adhere to a food budget.

### Intrapersonal influences

Intrapersonal influences included attitudes related to masculinity; food-related tasks and skills; nutrition knowledge and awareness, and moralising consumption of certain foods.

#### Attitudes related to masculinity

Men from both educational groups reported that they did not believe that preparing and consuming healthy food were negatively associated with principles of masculinity, but rather were important for good health. Some tertiary educated men thought perceptions that it was unmasculine to eat healthfully had become less common over time, while others from both groups thought eating healthfully actually enhanced masculinity.

“Things have changed. It might just be a reflection of my own friends, but I think a lot of guys I know cook more and want to eat a greater range of foods. I think there is a change where guys are picking up more responsibility at home.” Tertiary educated man.

“I tend to think if you eat healthy it would give you a greater sense of masculinity from a male point of view.” Non-tertiary educated man.

#### Food-related tasks and skills

Food-related tasks and skills were discussed as an influence on men’s eating behaviours by almost all men from both education groups. Tertiary educated men reported taking part in meal planning, food purchasing and preparation (although to a lesser degree than non-tertiary educated men), and adding extra vegetables to a dish to make it healthier. Some non-tertiary educated men described themselves as expert cooks, while others felt they had sufficient skills to put simple meals together. Both groups of men also frequently prepared their lunch for work.

“Tonight I’ve got leftover pasta… I just added frozen peas and some fresh asparagus, which I just boiled quickly and I added it in...” Tertiary educated man.

“If I do prepare a meal I might make myself some bacon and eggs on toast or I might make myself a burger if the materials are here at the time.” Non-tertiary educated man.

Men from both groups identified several reasons for cooking, including sharing the workload with their partner or spouse and/or because they enjoyed cooking. A few of the non-tertiary educated men described eating at home because it was cheaper to cook at home than to eat out. Some non-tertiary educated men also described sharing the food preparation workload due to time constraints, such that whoever in the household arrived home earliest after work, or had more time, did the cooking.

“[Dinner time is] the time that my wife sort of works a bit later and I’m working days and I’ve got time to cook ‘til she comes home… I like the taste and I like experimenting with cooking and making a few different things.” Non-tertiary educated man.

#### Nutrition knowledge and awareness

Men from both groups were aware of the importance of eating healthfully and thought people, particularly other men, were far more aware of the importance of eating healthfully than in the past, and that awareness was continuing to grow over time.

“I just think it’s a common theme about people my age, and my circle of people are always aware of our weight and know what we should be eating, but still have a taste for the not so healthy stuff.” Tertiary educated man.

“I’d say it’s highly likely [a man similar to me would regularly eat healthy food]. Because I think that’s the way society is going… because you see all the ads, you see all the cooking shows, and I think it’s just an education thing.” Non-tertiary educated man.

Both groups of men considered healthfulness when making food choices, with many choosing foods specifically because they felt they were healthy. Nutrition knowledge was not determined by skill-testing questions and men were not asked to directly compare their knowledge to other men’s knowledge, however, non-tertiary educated men perceived that they had lower nutrition-related knowledge than men with a tertiary education, and sometimes described misperceptions related to nutrition and body weight.

“Steaks are probably... better for me than any of the other fatty food. Even with sausages sometimes they can be real fatty where at least I know if a steak’s done properly there’s not much chance of a lot of fat still being inside of it”. Non-tertiary educated man.

“My understanding is that fat is only stored to a point and then your body won’t take anymore. What we assume is eating too much fat is actually carbohydrates stored as fat… In actual fact [people] are not fat. They’re just carrying an enormous amount of carbohydrate that they’re not using.” Non-tertiary educated man.

#### ‘Moralising’ consumption of certain foods

Men in both groups moralised consumption of certain foods based on their perceived healthfulness, particularly snack foods. In 1999, Rozin described moralisation as the act of accreting moral value to activities or objects (such as food) that were previously without moral value [52]. Moralising food consumption can be regarded as translating food judgements into corresponding behavioural rules. For example, men associated choosing ‘good’ food with good health or high self-control, while ‘bad’ food choices were linked with poor health and low self-control. Such food judgements can be taken further to imply that certain food choices are righteous/sinful, or moral/immoral [53]. Men in both groups often described healthy food as ‘right’ or ‘sensible’, while consumption of unhealthy foods was construed negatively, associated with feelings of guilt, or viewed as ‘terrible’.

“I always favour seafood because I tend to think it’s a more sensible choice… I think seafood’s invariably a healthy choice...” Non-tertiary educated man.

### Social influences

Social influences on eating behaviours identified included the influences of partners/spouses and children, and the perception that no-one influenced eating behaviours.

#### Partners and spouses

Among those men with partners, more of the tertiary educated men than those with non-tertiary education described believing that their partner had a healthy influence on their eating behaviours. In the majority of cases partners’ main mechanism of influence was by acting as gatekeepers of the home food environment by controlling the healthfulness of foods purchased, and preparing nutritious meals. Some tertiary educated men also thought their partners also verbally encouraged them to eat healthfully, or that their partner was a healthy role model.

“[My wife helps me eat more healthfully]… by positive reinforcement, by actively seeking and assisting in healthy choices, healthy recipes and healthy food” Tertiary educated man.

#### Children

Among both groups of men, most who had children thought their children influenced them to eat healthfully. A number of fathers described choosing healthier foods in order to make them available to their children, as well as to role-model healthy eating for their children.

“You’re kind of the biggest influence on [children], so it’s just more about leading by example... [I eat] probably healthier, because there’s a sort of a guilt associated with what you feed them… Some foods you choose because the children are watching.” Non-tertiary educated man.

#### No-one influences eating behaviour

Several non-tertiary educated men stated they did not believe anyone else exerted influence on their eating behaviours, despite many of these men having partners and/or children. This view was not identified by tertiary educated men.

“No-one [influences my eating behaviour]. I’m a single person. I’ve got my own business. No-one really influences me with food at all.” Non-tertiary educated man (lives alone).

“No-one. Just me... Because just when I first left home I was still living all the time on my own.” Non-tertiary educated man (lives alone).

“There’s no outside influences. No, not at all. I’m guided intuitively by historically what I could afford to eat many years ago and what I thought was the best choice because of what I could afford and that would be my sole reason.” Non-tertiary educated man (participant is married with children).

### Environmental influences

Environmental influences identified by both groups of men included availability of, and access to, healthy and unhealthy foods as well as convenience and cost.

#### Availability of and access to healthy and unhealthy foods

All men discussed availability of, and access to, healthy and unhealthy foods at home, work, and in the local neighbourhood as affecting food choice. Almost all men from both groups felt healthy food was readily available (e.g. where they did their weekly grocery shopping); and accessible in the local neighbourhood (e.g. at local markets and supermarkets that could be reached either on foot or by car in a short amount of time).

Tertiary educated men thought access to particular foods increased the likelihood those foods would be eaten, therefore ready access to healthy foods would result in eating more healthfully in general. A few non-tertiary educated men also chose foods at home, particularly snack foods, simply because they were readily accessible.

“If I’m in the right frame of mind when I’m shopping I’ll buy better things… I’ll buy more vegetables and more fruit… And if I buy it, I eventually will eat it. I don’t like wasting stuff… Just making sure that you buy more fruit and vegetables than you think you need… because they’re there, you can think of things you can do with them.” Tertiary educated man.

“[For snacks, I eat] anything I can get my hands on really. I’m a bit of a human garbage disposal, so there’s fruits and biscuits and nuts and whatever – chocolate. Anything I can get. Chips. Anything I can get a hold of. Anything in front of me.” Non-tertiary educated man.

Some non-tertiary educated men had mobile worksites, and so work lunch choices were influenced by what was available in the neighbourhood surrounding their workplace, i.e. they purchased food wherever they were located for a job.

“Not an actual workplace cafeteria. I’m self-employed. I’m sort of all over the place so it’d be just like a shop [where I buy my lunch when at work]. Yeah, just whatever’s closest.” Non-tertiary educated man.

#### Convenience

Almost all men from both groups cited convenience as a major influence of food choice, selecting foods – particularly breakfast and lunch foods – because they were close to hand, and quick to purchase and consume. Among men who purchased work lunches, several from both groups considered the convenience and time it took to access food influenced their choices, often leading to less healthy food purchases.

“There’s always a lot more temptation to eat junky food [for work lunch], because it’s really easy and it’s there, and it’s just about everywhere that you go. You can just grab it and eat it, you don’t have to think about it. And I’ve noticed if you have to wait and think about it, you generally change your mind.” Tertiary educated man.

#### Cost

Cost influenced men’s food choices. All tertiary educated men considered cost when choosing food, and the perception that healthy food was expensive was prominent among tertiary educated men, but not among non-tertiary educated men. Tertiary educated men thought the cost of healthy food was prohibitive when doing the grocery shopping, and unhealthy food items available in supermarkets were often cheap, or on special.

“It’s so much easier, in particular this country, to buy cheap take-away than it is to buy what’s often not so cheap healthy food and then do the groundwork of preparing. It’s easier and often cheaper... You walk into a supermarket and you’re going to pay AUS$3.00 for a bottle of [high-calorie beverage] and AUS$3.50 for a bottle of water. How is that possible?” Tertiary educated man.

Almost all non-tertiary educated men also considered price when choosing foods, with some households having to stay within a budget when they shopped for food.

“Generally [we cook] the cheaper cuts of meat, mince and sausages… because we’re on a budget.” Non-tertiary educated man.

Men from both groups talked about considering cost along with other influences when choosing foods. Consistently, the interplay between cost, convenience, taste, and healthfulness of foods were considered together before a choice was made. Among men from both groups, those who prioritised health tended to consider cost as a secondary influence after health, followed by convenience, with taste being less important; among those who did not prioritise health, cost and convenience were more important over health and taste considerations.

“Probably convenience, cost and health would be the main three [influences to consider when choosing lunch] for me. It’s just with my work and home life, [having a] schedule where we’re home, with the little one at lunch time [and] she’s having a sleep during my lunch [I choose what is convenient], and then other times cost. It’s more cost effective for me to take [my lunch to work with me], something that I like to eat rather than have to pay $8 for a salad roll when I can make one and bring one from home and don’t have to go looking for it as well.” Tertiary educated man.

“[Food] definitely has to be filling because the price of food these days out is usually expensive. Definitely filling… You need to be content. You don’t want to have one hot dog and go, ‘Gee, I’m still hungry.’ At the end of the day you might get to a place and there’s only two options [available]. So you look at that and convenience, what’s easy, what’s simple. Price does come into it. Again, it’s hard to judge because everything that you buy these days is pricey anyway.” Non-tertiary educated man.

## Discussion

The present investigation aimed to examine potential explanations of socioeconomic differences in men’s eating behaviours by qualitatively exploring influences on eating among men of tertiary and non-tertiary education levels. Salient themes among men from both education groups included influences from intrapersonal, social, and environmental domains. Influences more predominant among tertiary educated men included having more advanced food-related skills but relatively less involvement in food-related tasks compared non-tertiary educated men; partner/spouse support for healthy eating; access to healthy foods; and views relating to food cost. Prominent influences among men with non-tertiary education levels included having limited cooking skills (e.g. being able to prepare simple dishes with few steps and uncomplicated techniques) but more frequently being involved in food-related tasks, and perceiving having limited nutrition knowledge when compared with discussion by tertiary educated men. These men also identified more often that no-one influenced their diet; they had mobile worksites; and adhered to a food budget.

Neither group perceived food preparation or healthy eating to be at odds with the concept of masculinity, a finding which is divergent with those of previous studies that showed men, irrespective of education level or occupation, considered healthy eating as feminine [21, 54, 55]. It may be that with increasing global recognition of the importance of diet for chronic disease prevention, eating for good health has become more acceptable and normative among men since those earlier studies were published. Men’s perceptions about masculinity described in the present investigation may also be attributed to workforce and societal changes in women and careers, with fewer men being the family’s primary income provider, and with fewer women staying home to perform all food-related tasks than previously. Further, the majority of participants in the present investigation were aged ≥45 years, and may have greater awareness of the importance of health behaviours as they age and face increased risk of diet-related disease.

When discussion about food-related tasks and skills was examined, tertiary educated men’s cooking skills were more developed, but they had less involvement in food-related tasks than non-tertiary educated men who had more limited cooking skills but regular involvement in food-related tasks. These findings correspond with those reported previously. For example, low income US men were nearly three times more likely to be involved in meal planning and preparation compared to their wealthier counterparts [56], and Norwegian men working in blue collar occupations (carpenters) were more likely to share food shopping and preparation with their partner/spouse compared to men in white collar occupations (engineers) [57]. Consistent with our findings regarding education level and cooking skills, when self-described cooking skills were compared between Swiss men, those with high education levels had more elaborate cooking skills than less educated men [58].

Social influences on men’s eating behaviours included those in their family unit (i.e. partner/spouse, and/or children), or, as for several non-tertiary educated men, no other individuals. Partner/spousal support for healthy eating was recognised as important by tertiary educated men in our study, but not among those with non-tertiary education. Conversely, low income British men previously identified female figures (e.g. spouses/partners, mothers, grandmothers) as positive influences on their eating behaviours [59]. Similarly, Dutch men with lower vocational education or below stated they would eat healthfully if their spouse/partner did [60]. A previous Australian nutrition and physical activity intervention incorporating social support by partners resulted in significant decreases in total and saturated fat consumption, and significant increases in fibre intake among men and women [61], implying that greater social support from spouses/partners would encourage men to eat more healthfully. It is unclear why our findings diverged from these previous studies, however it may simply be a function of studying different samples. Fathers from both education groups acknowledged the importance of role-modelling healthy eating for their children, and how this encouraged their own healthy eating. Previous research showed that Australian children’s total fruit consumption was positively associated with that of their father [62], and thus supports observations in the present investigation. That some non-tertiary educated men in the present investigation thought no-one influenced their diet was novel, and contradictory to previous research suggestive that social support for healthy eating encouraged less educated or low income men to adhere to healthier eating behaviours [59, 60]. On balance, findings from the present investigation and previous research suggest that role-modelling and social support are important factors for supporting men to eat healthfully, and have the potential to be powerful mechanisms through which improvements in men’s diets could be achieved if incorporated into future nutrition promotion initiatives, for example, engaging men along with their partners in intervention strategies including nutrition education and cooking classes.

Tertiary educated men in our study considered healthy foods to be expensive; however, although non-tertiary educated men reported having to adhere to a food budget, they did not generally describe healthy foods as expensive. Potential explanations for this paradoxical finding could be that only six of the non-tertiary educated men had low income and may have been able to afford healthy foods. However, previous research among socioeconomically disadvantaged men showed they did not consider healthy foods prohibitively expensive [59, 60]. The present investigation also revealed that men chose foods by considering a number of influences in conjunction at multiple socioecological levels (e.g. cost, taste, etc.). The observed interplay between influences on men’s eating behaviours implies multiple factors shape men’s dietary behaviours. It also suggests employing a qualitative approach to explore influences on men’s eating behaviours across the domains of the social ecological model in unison, such as employed in the present investigation is advantageous. This can yield a deeper understanding of how influences across domains interact and can be utilised in future to further inform research and interventions aimed at improving men’s eating behaviours.

Factors identified as potential influences on socioeconomic inequalities in men’s diets in this study need confirmation in larger samples using quantitative methods. Acknowledging this, the present investigation has elucidated key levers that could, if confirmed, be targeted in initiatives aimed at reducing inequalities in eating behaviours, in turn ameliorating the socioeconomic ‘gap’ and adverse health and economic outcomes associated with these inequalities. For example, strategies to promote healthy eating among non-tertiary educated men could focus on developing greater nutrition knowledge, improving cooking skills, identifying key social supports for healthy eating, and providing skills and strategies to purchase healthy foods, particularly whilst at work, whether at a fixed or mobile worksite, and on a budget. Strategies that could support tertiary educated men to eat healthily could include promoting greater involvement in food-related tasks and education about choosing low cost healthy foods. Previous programs incorporating some strategies identified above have successfully promoted healthy eating among women and men [63] including those experiencing socioeconomic disadvantage [64]. However, given challenges in engaging men in such programs [65], policy and practice should not only focus on developing nutrition promotion initiatives aimed at improving men’s diet that are custom-made to specific socioeconomic groups, but also incorporate specific tailoring to engage men.

Study limitations should be acknowledged. Participating men may have been more interested in nutrition and health than non-participants, resulting in possible participation bias. Transferability of findings may be limited by a single measure of SEP being used to define the sample. Almost all participating men were employed, and had professional occupations; and only half of non-tertiary educated men had low incomes. More sensitive measures of education (beyond the binary categorisation applied in the present investigation) could be considered in future research. Further, SEP is not determined by education alone, and is only one of many possible measures, when in fact SEP is best described in a more complex way by considering multiple factors such as income, education, and occupation simultaneously, not singly. As no data about men’s ethnicity or culture were gathered in the present investigation, it was not possible to make any observations about possible cultural variations in views between men. Exploring cultural differences in conjunction with socioeconomic differences may be considered in future. Also, as more than half of participants were aged 45–54 years, the generalisability to men of other age groups may be limited. Men who identified as having a partner were not asked to disclose the sex of their partner. It is unclear if study findings would vary whether the couple was same-sex or opposite-sex, and is therefore acknowledged as a limitation. Nevertheless, qualitative studies do not intend to focus on general sample representativeness, but rather aim to generate a range of responses and hypotheses for potential follow up in future research [38].

Men may also have provided socially desirable responses, such as stating they had more favourable eating behaviours than in reality, yet participants also identified challenges faced in consuming healthy foods and openly discussed barriers to doing so, suggesting that socially desirable responses were minimised. Further, participants’ responses might have been influenced by being interviewed by another male; views presented may have inadvertently been driven by participants’ perceptions of shared masculine identity with, or reciprocal enactment of masculinity by the male interviewer, and consequently resulting in a more idealised cultural notion of masculinity [41]. However, as this was not reflected in the responses observed (e.g. healthy eating was not perceived to be unmasculine), the use of male interviewers here could be interpreted as a strength as there may have been reciprocity between the interviewer and interviewee, resulting in richer data than may have been gathered by female interviewers [41]. Also, using a one-on-one telephone interview methodology may have reduced some response bias as participants may have been less affected by cues from facial expressions or perceived social desirability from the researcher, e.g. in face-to-face interviews, or other participants, e.g. in a focus group setting [66, 67]. While using a telephone method also has disadvantages, including lack of visual cues and difficulty building rapport [68], this method was deemed necessary as participants were recruited across a wide geographical area. Finally, data analysis occurred after data collection was complete, and therefore emerging themes could not be checked during the data collection process.

Study strengths include the qualitative design which provided in-depth, comprehensive insights into socioeconomic differences in influences on men’s eating behaviours, with perspectives provided by men living in two regions of Australia, drawn from different educational strata. A further notable strength of the study is that it provided unique insights into men’s eating behaviours overall, irrespective of SEP.

## Conclusions

To conclude, the present investigation provided insights into individual, social and environmental influences on the eating behaviours of men with divergent education levels, expanding the knowledge base around this important topic. Key potential drivers of socioeconomic inequalities in men’s eating behaviours were identified, with potential to inform novel strategies to encourage men to eat healthfully. Future quantitative research is required to examine how factors identified in the present investigation are associated with men’s dietary intakes across socioeconomic strata; how they might explain socioeconomic differences in men’s diets; and the feasibility of adopting various strategies to support healthy eating among men in different socioeconomic groups.

## Additional file

Additional file 1: **Table S1.** Semi-structured interview questions investigating ns on men’s eating behaviours. Summary of semi-structured interview questions used in the present investigation. (DOCX 27 kb)

## Abbreviation

SEP: Socioeconomic position

## References

[1] Charlton K, Kowal P, Soriano MM, Williams S, Banks E, Vo K (2014). Fruit and vegetable intake and body mass index in a large sample of middle-aged Australian men and women. Nutrients.

[2] Prattala R, Paalanen L, Grinberga D, Helasoja V, Kasmel A, Petkeviciene J (2007). Gender differences in the consumption of meat, fruit and vegetables are similar in Finland and the Baltic countries. Eur J Pub Health.

[3] Seguin RA, Aggarwal A, Vermeylen F, Drewnowski A (2016). Consumption frequency of foods away from home linked with higher body mass index and lower fruit and vegetable intake among adults: a cross-sectional study. J Environ Public Health.

[4] Australian Bureau of Statistics (2014). Australian Health Survey: Nutrition First Results - Foods and Nutrients, 2011–12. Report number: 4364.0.55.007.

[5] Smith KJ, Blizzard L, McNaughton SA, Gall SL, Dwyer T, Venn AJ (2012). Takeaway food consumption and cardio-metabolic risk factors in young adults. Eur J Clin Nutr.

[6] Benjamin EJ, Blaha MJ, Chiuve SE, Cushman M, Das SR, Deo R (2017). Heart disease and stroke Statistics-2017 update: a report from the American Heart Association. Circulation.

[7] Non-Communicable Diseases Risk Factor Collaboration (NCD-RisC) (2016). Worldwide trends in diabetes since 1980: a pooled analysis of 751 population-based studies with 4.4 million participants. Lancet.

[8] Australian Institute for Health and Welfare (2012). Australia’s Health 2012. Report number: AUS 156.

[9] Giskes K, Avendano M, Brug J, Kunst AE (2010). A systematic review of studies on socioeconomic inequalities in dietary intakes associated with weight gain and overweight/obesity conducted among European adults. Obes Rev.

[10] Giskes K, van Lenthe F, Avendano-Pabon M, Brug J (2011). A systematic review of environmental factors and obesogenic dietary intakes among adults: are we getting closer to understanding obesogenic environments?. Obes Rev.

[11] Ball K, Lamb KE, Costa C, Cutumisu N, Ellaway A, Kamphuis CB (2015). Neighbourhood socioeconomic disadvantage and fruit and vegetable consumption: a seven countries comparison. Int J Behav Nutr Phys Act.

[12] Giskes K, Turrell G, Patterson C, Newman B (2002). Socioeconomic differences among Australian adults in consumption of fruit and vegetables and intakes of vitamins A, C and folate. J Hum Nutr Diet.

[13] Miura K, Giskes K, Turrell G (2012). Socio-economic differences in takeaway food consumption among adults. Public Health Nutr.

[14] Galobardes B, Shaw M, Lawlor DA, Lynch JW, Davey SG (2006). Indicators of socioeconomic position (part 1). J Epidemiol Community Health.

[15] Stokols D (1996). Translating social ecological theory into guidelines for community health promotion. Am J Health Promot.

[16] Thornton LE, Pearce JR, Ball K (2014). Sociodemographic factors associated with healthy eating and food security in socio-economically disadvantaged groups in the UK and Victoria, Australia: Cambridge University Press.

[17] Motteli S, Siegrist M, Keller C (2017). Women’s social eating environment and its associations with dietary behavior and weight management. Appetite.

[18] Wang Da-Hong, Kogashiwa Michiko, Mori Naoko, Yamashita Shikibu, Fujii Wakako, Ueda Nobuo, Homma Hiroto, Suzuki Hisao, Masuoka Noriyoshi (2016). Psychosocial Determinants of Fruit and Vegetable Consumption in a Japanese Population. International Journal of Environmental Research and Public Health.

[19] Kegler MC, Alcantara I, Haardorfer R, Gazmararian JA, Ballard D, Sabbs D (2014). The influence of home food environments on eating behaviors of overweight and obese women. J Nutr Educ Behav.

[20] Thornton LE, Lamb KE, Ball K (2013). Employment status, residential and workplace food environments: associations with women’s eating behaviours. Health Place.

[21] Roos G, Prattala R, Koski K (2001). Men, masculinity and food: interviews with Finnish carpenters and engineers. Appetite.

[22] von Bothmer MI, Fridlund B (2005). Gender differences in health habits and in motivation for a healthy lifestyle among Swedish university students. Nurs Health Sci.

[23] Courtenay WH (2000). Constructions of masculinity and their influence on men’s well-being: a theory of gender and health. Soc Sci Med.

[24] Gough B (2013). The psychology of men's health: maximizing masculine capital. Health Psychol.

[25] Pampel FC, Krueger PM, Denney JT (2010). Socioeconomic disparities in health behaviors. Annu Rev Sociol.

[26] Girois SB, Kumanyika SK, Morabia A, Mauger E (2001). A comparison of knowledge and attitudes about diet and health among 35 to 75 year-old adults in the United States and Geneva, Switzerland. Am J Public Health.

[27] Morgan PJ, Warren JM, Lubans DR, Collins CE, Callister R (2011). Engaging men in weight loss: experiences of men who participated in the male only SHED-IT pilot study. Obes Res Clin Pract.

[28] Furey S, McIlven H, Strugnell C (2000). Cooking skills: a diminishing art?. Nutr Food Sci.

[29] du Plessis K (2012). Factors influencing Australian construction industry apprentices’ dietary behaviors. Am J Mens Health.

[30] Maguire ER, Burgoine T, Penney TL, Forouhi NG, Monsivais P (2017). Does exposure to the food environment differ by socioeconomic position? Comparing area-based and person-centred metrics in the Fenland study, UK. Int J Health Geogr.

[31] Smith LH, Holm L (2011). Obesity in a life-course perspective: an exploration of lay explanations of weight gain. Scand J Public Health.

[32] Tong A, Sainsbury P, Craig J (2007). Consolidated criteria for reporting qualitative research (COREQ): a 32-item checklist for interviews and focus groups. Int J Qual Health Care.

[33] Van Dyke N, Maddern CM, Walker R, Reibel T (2014). Young people’s experiences with health services – Final report. Report number: Perth, Western Australia.

[34] Van Dyke N (2009). Methodological issues in the design and conduct of public health computer assisted telephone interview surveys: the case of informal carers in Australia. Aust J Prim Health.

[35] Jezewska-Zychowicz Marzena, Gębski Jerzy, Guzek Dominika, Świątkowska Monika, Stangierska Dagmara, Plichta Marta, Wasilewska Milena (2018). The Associations between Dietary Patterns and Sedentary Behaviors in Polish Adults (LifeStyle Study). Nutrients.

[36] Sakurai M, Nakagawa H, Kadota A, Yoshita K, Nakamura Y, Okuda N (2018). Macronutrient intake and socioeconomic status: NIPPON DATA2010. J Epidemiol.

[37] Wang DD, Leung CW, Li Y, Ding EL, Chiuve SE, Hu FB (2014). Trends in dietary quality among adults in the United States, 1999 through 2010. JAMA Intern Med.

[38] Tolley EE (2016). Qualitative methods in public health: a field guide for applied research.

[39] Galobardes B, Shaw M, Lawlor DA, Lynch JW, Davey SG (2006). Indicators of socioeconomic position (part 2). J Epidemiol Community Health.

[40] Payette H, Shatenstein B (2005). Determinants of healthy eating in community-dwelling elderly people. Can J Public Health.

[41] Schwalbe M, Wolkomir M (2001). The masculine self as problem and resource in interview studies of men. Men Masculinities.

[42] Ritchie J, Lewis J, McNaughton Nicholls C, Ormston R (2014). Qualitative research practice: a guide for social science students and researchers., second edn.

[43] Parmenter K, Waller J, Wardle J (2000). Demographic variation in nutrition knowledge in England. Health Educ Res.

[44] McDermott AJ, Stephens MB (2010). Cost of eating: whole foods versus convenience foods in a low-income model. Fam Med.

[45] Ralph L, Seaman CEA, Woods M (1996). Male attitudes towards healthy eating. Brit Food J.

[46] Ball K, Timperio A, Crawford D (2009). Neighbourhood socioeconomic inequalities in food access and affordability. Health Place.

[47] Sandelowski M (2000). Whatever happened to qualitative description?. Res Nurs Health.

[48] Sandelowski M (2010). What's in a name? Qualitative description revisited. Res Nurs Health.

[49] Green J, Willis K, Hughes E, Small R, Welch N, Gibbs L (2007). Generating best evidence from qualitative research: the role of data analysis. Aust N Z J Public Health.

[50] Lipscomb M (2012). Abductive reasoning and qualitative research. Nurs Philos.

[51] Barbour RS (2001). Checklists for improving rigour in qualitative research: a case of the tail wagging the dog?. BMJ.

[52] Rozin P (1999). The process of moralization. Psychol Sci.

[53] Askegaard S, Ordabayeva N, Chandon P, Cheung T, Chytkova Z, Cornil Y (2014). Moralities in food and health research. J Mark Manag.

[54] Kelly A, Ciclitira K (2011). Eating and drinking habits of young London-based Irish men: a qualitative study. J Gend Stud.

[55] Gough B, Conner MT (2006). Barriers to healthy eating amongst men: a qualitative analysis. Soc Sci Med.

[56] Harnack L, Story M, Martinson B, Neumark-Sztainer D, Stang J (1998). Guess who's cooking? The role of men in meal planning, shopping, and preparation in US families. J Am Diet Assoc.

[57] Wandel M, Roos G (2005). Work, food and physical activity. A qualitative study of coping strategies among men in three occupations. Appetite.

[58] Hartmann C, Dohle S, Siegrist M (2013). Importance of cooking skills for balanced food choices. Appetite.

[59] Daborn C, Dibsall L, Lambert N (2005). Understanding the food related experiences and beliefs of a specific group of low-income men in the UK. Health Educ.

[60] Romeike K, Abidi L, Lechner L, de Vries H, Oenema A (2016). Similarities and differences in underlying beliefs of socio-cognitive factors related to diet and physical activity in lower-educated Dutch, Turkish, and Moroccan adults in the Netherlands: a focus group study. BMC Public Health.

[61] Burke V, Mori TA, Giangiulio N, Gillam HF, Beilin LJ, Houghton S (2002). An innovative program for changing health behaviours. Asia Pac J Clin Nutr.

[62] Hall L, Collins CE, Morgan PJ, Burrows TL, Lubans DR, Callister R (2011). Children’s intake of fruit and selected energy-dense nutrient-poor foods is associated with Fathers’ intake. J Am Diet Assoc.

[63] Thomson CA, Ravia J (2011). A systematic review of behavioral interventions to promote intake of fruit and vegetables. J Am Diet Assoc.

[64] Bull ER, Dombrowski SU, McCleary N, Johnston M (2014). Are interventions for low-income groups effective in changing healthy eating, physical activity and smoking behaviours? A systematic review and meta-analysis. BMJ Open.

[65] Gavarkovs AG, Burke SM, Petrella RJ (2016). Engaging men in chronic disease prevention and management programs: a scoping review. Am J Mens Health.

[66] Musselwhite K, Cuff L, McGregor L, King KM (2007). The telephone interview is an effective method of data collection in clinical nursing research: a discussion paper. Int J Nurs Stud.

[67] Marcus AC, Crane LA (1986). Telephone surveys in public health research. Med Care.

[68] Carr ECJ, Worth A (2001). The use of the telephone interview for research. J Res Nurs.

