# Supplementary material for: A qualitative study of the drivers of socioeconomic inequalities in men’s eating behaviours
Source: BMC Public Health. 2018 Nov 14;18:1257. doi: 10.1186/s12889-018-6162-6 (PMC6236940; doi:10.1186/s12889-018-6162-6)
Supplement: Supplementary file 1 — Table S1. Semi-structured interview questions investigating ns on men’s eating behaviours. Summary of semi-structured interview questions used in the present investigation. (DOCX 27 kb) [file 12889_2018_6162_MOESM1_ESM.docx]

**Table S1** Semi-structured interview questions investigating influences on men’s eating behaviours

|  | **Interview questions** |
| --- | --- |
| **Food task responsibilities** | “Who purchases and prepares most of the food in your household?”  “Why do you OR does [person named above] do most of this?” |
|  |  |
|  | “How many of those nights do you normally do the cooking for dinner?”  “Why do you normally do the dinner cooking [number] of times per week?” |
|  |  |
| **Influences on eating behaviours** |  |
| *Breakfast/ Lunch/ Dinner/ Snacks* | “What do you think are the main things that influence what you eat and drink at breakfast/ lunch/ dinner/ for snacks?” |
|  |  |
| *Further lunch questions* | “Does what you have for lunch vary much depending on whether it’s a weekday or a weekend?”  IF there is variation:  “What’s the difference?” |
|  |  |
|  | “I’d now like you to imagine that you are buying your lunch. I’m going to read out a short list of things that might affect what you choose. Can you please tell me which of these is the most important? Would it be… [rotate options] ‘Taste’, ‘Price’, ‘How healthy it is’, ‘How long it takes to get it’, ‘How long it takes to eat it’, ‘What your co-workers or others around you are eating’, or ‘What’s available’?”  IF APPLICABLE:  “Which would be the second/third most important?” |
|  |  |
|  | “In a typical week, how many days is your lunch…’Eaten at home or brought to work from home’, ‘Bought at a workplace cafeteria’, ‘Eaten at a café or restaurant (eaten in)’, ‘Takeaway’, ‘No particular pattern – varies from week to week’, and ‘Other (Specify)’ ”  “Why is there this pattern?” OR “Why do you have no particular pattern with your lunch?” |
|  |  |
| **Influences on eating choices: trade-offs between health, convenience, peer modelling, price, accessibility, taste** | IF PARTICIPANT IDENTIFIED ‘how long it takes to eat it’ as the most important consideration when choosing lunch:  “I’d now like you to imagine that you are buying your lunch and you have two options. Would you choose a [healthy option of participant’s choosing] if it took you 5 minutes to eat it or an [unhealthy option of participant’s choosing] if it took you 20 minutes to eat it?”  “What if the time it took to eat your lunch were reversed? So would you choose the [healthy option] if it took you 20 minutes to eat it or the [unhealthy option] if it took you 5 minutes to eat it?”  IF PARTICIPANT IDENTIFIED ‘what co-workers/others around you are eating’ as the most important consideration when choosing lunch:  “I’d now like you to imagine that you are buying your lunch and you have two options. If most of your co-workers or others around you were eating a [healthy option of participant’s choosing], would you choose a [healthy option] or a [unhealthy option participant’s choosing]?”  “If most of your co-workers or others around you were eating a [unhealthy option], would you choose a [healthy option] or a [unhealthy option]?” |
|  |  |
|  | FURTHER QUESTIONS: |
|  | “I’d now like you to imagine that you are buying your lunch and you have two options. Would you choose a [healthy option] at AUS$6 or a [unhealthy option] at AUS$10?” |
|  |  |
|  | “What if the prices were reversed? So would you choose the [healthy option] at AUS$10 or the [unhealthy option] at AUS$6?” |
|  |  |
|  | “What if the [healthy option] costs AUS$5 and the [unhealthy option] costs AUS$12? Which one would you be more likely to buy?” |
|  |  |
|  | “What if the prices were reversed? So would you choose the [healthy option] at AUS$12 or the [unhealthy option] at AUS$5?” |
|  |  |
|  | “What if you have to walk 10 minutes to get the [healthy option], but the [unhealthy option] is available right next to wherever you are. Which one would you be more likely to buy?” |
|  |  |
|  | “What about the reverse? What if the [healthy option] is right next to where you are but to have the [unhealthy option], you have to walk 10 minutes. Which one would you be more likely to buy?” |
|  |  |
|  | “Next I’d like you to think of an example of a healthy lunch that doesn’t taste as good as the [unhealthy option]. It shouldn’t be a lunch that you hate – just one that you don’t like as much as [healthy option]. Maybe something you know is good for you but you don’t really love. What might this be?” |
|  |  |
|  | “Let’s assume that the following two lunches cost the same and can be bought from the same shop. So now the two choices are [second healthy option] and [unhealthy option]. Which one would you be more likely to buy?” |
|  |  |
| **Social influences on eating choices** | “Other than yourself, who would you say has the biggest influence on what you eat and drink?”  IF NOT RESPONDED TO AS ‘no-one’:  “Would you say that [person named above] would generally lead you to eat healthier or unhealthier? How would [this person] do this?” |
|  |  |
|  | “Is there anyone else who has a big influence on what you eat and drink?”  IF APPLICABLE, repeat above questions for second most important influential person, and third most important person. |
| **Neighbourhood availability** | “Some of the types of foods generally considered to be healthy include fruit, vegetables, fish and lower-fat meats, and whole grains.  I’d like you to think about how easy or difficult it is for you to buy healthy food like this in your neighbourhood. On a scale of 1 – ‘Impossible’ to 10 – ‘Very easy’, how easy or difficult is it for you to buy healthy food in your neighbourhood?”  IF 1-9:  “Can you tell me why you said [1-9] rather than a higher number?”  IF 2-10:  “Can you tell me why you said [2-10] rather than a lower number?” |
|  |  |
| **Body weight** | “Would you say you are… ‘Underweight’, ‘Normal or average weight’, ‘Overweight’ or ‘Very overweight or obese’ ” |
|  |  |
|  | IF PARTICIPANT provided answer to weight status question:  “Do you think that being [weight status] has an influence on the foods and drinks you consume?”  IF ‘yes’:  “In what ways? How would you say being [weight status] has an influence on the foods you eat?” |
|  |  |
| **Other influences** | “Other than things we’ve already discussed, is there anything else you think influences what you eat and drink?” |
|  |  |
| **Perceptions of other men’s eating behaviours (social norms) and masculinity** | “Now I’d like you to imagine a man who is similar to yourself in lots of ways – same age, same education, [similar household structure], living in the same neighbourhood. On a scale of 1 – ‘Very unlikely’ to 10 – ‘Very likely’, how likely do you think it is that he’d regularly eat healthy food for lunch?”  “Why did you give a reply of [number] out of 10?” |
|  |  |
|  | “If [the similar man] did regularly eat a healthy lunch, do you think his workmates or friends might think this was odd or unusual or perhaps make mention of it?”  IF ‘yes’:  “Do you think it would be very likely or somewhat likely?”  IF ‘no’:  “Do you think it would be somewhat unlikely or very unlikely?” |
|  |  |
|  | “Still thinking about [the similar] man, on a scale of 1 – ‘Very unlikely’ to 10 – ‘Very likely’, how likely do you think it is that he’d eat unhealthy food for lunch on a regular basis?”  “Why did you give a reply of [number] out of 10?” |
|  | “If [the similar man] did eat an unhealthy lunch on a regular basis, do you think his workmates or friends might think this was odd or unusual or perhaps make mention of it?”  IF ‘yes’:  “Do you think it would be very likely or somewhat likely?”  IF ‘no’:  “Do you think it would be somewhat unlikely or very unlikely?” |
|  |  |
|  | “Now I’d like you to imagine a different man. He is also about the same age as you, [similar household structure] and lives in your same neighbourhood, but he [has less than 12 years of education and works as a tradie (tradesman) OR has a tertiary degree and holds a professional job in an office]. On a scale of 1 – ‘Very unlikely’ to 10 – ‘Very likely’, how likely do you think it is that **this man** would regularly eat **healthy** food for lunch?”    “Why did you give a reply of [number] out of 10?” |
|  |  |
|  | “If [the different man] did eat a healthy lunch on a regular basis, do you think his workmates or friends might think this was odd or unusual or perhaps make mention of it?”  IF ‘yes’:  “Do you think it would be very likely or somewhat likely?”  IF ‘no’:  “Do you think it would be somewhat unlikely or very unlikely?” |
|  |  |
|  | “Still thinking about [the different man], on a scale of 1 – ‘Very unlikely’ to 10 – ‘Very likely’, how likely do you think it is that he’d eat unhealthy food for lunch on a regular basis?”  “Why did you give a reply of [number] out of 10?” |
|  |  |
|  | “If [the different man] did eat an unhealthy lunch on a regular basis, do you think his workmates or friends might think this was odd or unusual or perhaps make mention of it?”  IF ‘yes’:  “Do you think it would be very likely or somewhat likely?”  IF ‘no’:  “Do you think it would be somewhat unlikely or very unlikely?” |
|  |  |
|  | “Some people might say that there’s something odd or not masculine about trying to eat healthy foods. What do you think?” |
|  |  |
|  | “Can you think of a few reasons why men like you might want to eat healthfully/ might NOT want to eat healthfully?” |
|  |  |
| **Strategies to support healthy eating** | “What are some things that could be done that would help you eat more healthfully?” |
|  | IF PARTICIPANT only mentioned changes they could make to their own behaviours or attitudes:  “What about things other people could do, or maybe changes to the environment around you, that could help you eat more healthfully?” |
|  |  |
|  | “Of those things you just mentioned, which would you say is the most important?”  IF APPLICABLE:  “Which would be the second/third most important?” |
|  |  |
| **Closing** | “Is there anything else you’d like to tell us about the foods you like to eat and why you choose these foods?” |
